# A single centre experience of patients with rare cancers referred for early phase clinical trials

**DOI:** 10.1186/s12885-025-13934-2

**Published:** 2025-03-28

**Authors:** Angelos Angelakas, Natalie Cook, Donna M. Graham, Matthew Krebs, Fiona Thistlethwaite, Louise Carter

**Affiliations:** 1 Division of Cancer Sciences, Faculty of Biology, Medicine and Health, University of Manchester, Manchester, UK; 2 Department of Medical Oncology, The Christie NHS Foundation Trust, Manchester, UK

**Keywords:** Rare cancers, Early phase trials, Real world data, Prognosis, Precision medicine, Molecular profiling, Targeted treatment

## Abstract

**Background:**

Cancers affecting < 6/100,000/year are classified as rare, but they account for up to 25% of all cancers and are associated with worse 5-year survival than common cancers. Early-phase clinical trials (EPCTs) may represent a viable treatment option for patients with rare cancers as they have evolved significantly with novel designs and the increasing use of precision medicine.

**Methods:**

A retrospective study of patients with rare cancers referred to a large EPCT team at a UK specialist centre over 5 years (2016–2020) was conducted. Patient demographics, medical and oncological history, genomic variants, EPCT participation, responses and survival outcomes were analysed.

**Results:**

In total, 240 patients with rare cancers were included. The mean age at diagnosis was 51.7 years (range 16–84), 54.2% of the patients were female. The most frequent rare cancers originated from the digestive system (27.1%), female genital tract (20%) and head and neck (H + N) (18.3%). Molecular profiling was offered to 45.5% of the population, median number of gene alterations was 3 per patient (range 1–20) while actionable gene alterations were reported in 60.2% (*n* = 50) of those with identified gene aberrations. Fifty-one patients participated in EPCTs, with 39.2% achieving SD and 11.8% PR. Median PFS for trial participants was three months (95% CI 1.12 – 4.88) while median OS in the trial patients was 16 months (95% CI 9.10 – 22.90) compared to 7 months for non-trial participants (95% CI 5.50 – 8.51). Finally, poor Royal Marsden Hospital (RMH) prognostic score (2–3) was correlated with worse survival when controlling for age and sex (HR 1.714, 95% CI 1.19 – 2.46, *p* = 0.004).

**Conclusions:**

Participation of patients with rare cancers in EPCTs may be associated with a survival benefit and lead to the development of new treatments for these patients. Moreover, expanded use of precision medicine is paramount as it can inform targeted treatment selection in this heterogenous group.

**Supplementary Information:**

The online version contains supplementary material available at 10.1186/s12885-025-13934-2.

## Background

Rare cancers are a group of heterogeneous primary tumours which, although individually rare, collectively represent a significant proportion of all cancer diagnoses. In Europe, the United States (US) and the United Kingdom, rare cancers are estimated to account for 20%−24% of all cancers [1–4]. Developing an accepted definition for rare cancers has been challenging. However, in 2008, the Surveillance of Rare Cancers in Europe (RARECARE) project proposed that rare cancers should be defined as those with a crude incidence of less than 6 cases per 100,000 per year [3]. RARECARE identified 198 rare cancers according to this definition in the most recent update (2015) based on a 3-tier system [1, 3]. Tier-3 (bottom) includes the WHO names and ICD-O-3^9^ codes of distinctive cancer diagnoses. These were then grouped in tier-2 (middle) entities that involve similar therapeutic approaches and research. Finally, tier-2 categories were grouped in tier-1 (top) general entities requiring similar referral pathways and clinical competence [3]. For example, neuroendocrine tumours are a tier-1 diagnosis, endocrine carcinoma of the thyroid gland is a tier-2 diagnosis, and medullary carcinoma is a tier-3 diagnosis. In 2016, the Joint Action on Rare Cancers created a list of 12 major families of rare cancers based on the tier-1 categories mentioned above (Table 1) [5].

**Table 1:** The 12 major families of rare cancers developed by the Joint Action on Rare Cancers and the relevant tier-1 categories. Reproduced by Casali and Trama (2020) [5]

Major rare cancer families	Tier-1 categories
Head and neck tumours	• Epithelial tumours of the larynx
	• Epithelial tumours of the hypopharynx
	• Epithelial tumours of the nasal cavity and sinuses
	• Epithelial tumours of the nasopharynx
	• Epithelial tumours of major salivary glands and salivary gland type tumours
	• Epithelial tumours of the oropharynx
	• Epithelial tumours of the oral cavity and lip
	• Epithelial tumours of the eye and adnexa
	• Epithelial tumours of the middle ear
Digestive system tumours	• Epithelial tumours of the small intestine
	• Epithelial tumours of the anal canal
	• Epithelial tumours of the gallbladder and extrahepatic biliary duct
Thoracic tumours	• Epithelial tumours of the trachea
	• Thymomas and thymic carcinomas
	• Malignant mesothelioma
Female genital tumours	• Non- epithelial tumours of the ovary
	• Epithelial tumours of the vulva and vagina
	• Trophoblastic tumours of the placenta
Male genital and urogenital tumours	• Tumours of the testis and paratestis
	• Epithelial tumours of penis
	• Extragonadal germ cell tumours
	• Epithelial tumours of renal pelvis, ureter, and urethra
Skin cancers and non-cutaneous melanoma	• Mucosal melanoma
	• Uveal melanoma
	• Adnexal skin carcinomas
	• Kaposi sarcoma
Sarcomas	• Soft tissue sarcoma
	• Bone sarcoma
	• Gastrointestinal stromal tumours
Neuroendocrine tumours (NET)	• NET gastrointestinal pancreatic
	• NET lung
	• NET other sites
Endocrine organ tumours	• Thyroid cancers
	• Parathyroid cancer
	• Adrenal cortex cancer
	• Pituitary gland cancer
Central nervous system (CNS) tumours	• Glial tumours and others
	• Malignant meningioma
	• Embryonal tumours of CNS
Paediatric tumours	• Hepatoblastoma
	• Neuroblastoma and ganglioneuroblastoma
	• Nephroblastoma
	• Odontogenic malignant tumours
	• Olfactory neuroblastoma
	• Pancreatoblastoma
	• Pleuropulmonary blastoma
	• Retinoblastoma
Haematological tumours	• Lymphoid malignancies
	• Myelodysplastic syndromes
	• Myeloproliferative neoplasms (including mastocytosis)
	• Myelodysplastic/myeloproliferative neoplasms
	• Myeloid/ lymphoid neoplasms with eosinophilia and abnormalities of PDGFRA (platelet-derived growth factor receptor alpha), PDGFRB (platelet-derived growth factor receptor beta), or FGFR1 (fibroblast growth factor receptor 1), or with PCM1- JAK2
	• Acute myeloid leukaemia and related neoplasms

The incidence of rare cancers has steadily risen by 0.5% per year (1999–2007) which can be attributed to improved histopathological diagnosis as well as increased exposure to some risk factors such as HPV for anal and oropharyngeal cancers [1]. In the European Union, 650,000 new rare cancers were diagnosed yearly, while the total crude incidence rate was 115 per 100,000 per year. Haematological malignancies, head and neck cancers, female genital and digestive system tumours shared the highest incidence rates. In terms of survival, the 5-year relative survival was 49% in 2005–2007, significantly lower than that seen in common cancers (63%) [1]. In the US, 5-year relative survival was slightly higher compared to the EU, although still lower than common cancers in males (55% vs 75%) and females (60% vs 74%) [2].

Unfortunately, the atypical presentation and the individually low incidence of rare cancers have significantly limited the number of standard-of-care treatments developed for this population, contributing to the poorer survival outcomes [6, 7]. The contrast with common cancers is striking. For example, in the US, prostate and breast cancer share more than 20 category-one interventions, according to the National Comprehensive Cancer Network (NCCN), whereas there are no category-one interventions for chondrosarcomas [7].

Research has also faced significant barriers in developing novel treatment agents for rare cancers. Commercial sponsors more frequently invest in common cancers given the larger patient population and, therefore, the ease of delivering adequately powered studies with associated stronger data and the eventual larger potential market for the novel agent [6]. In contrast, for rare tumours, there are smaller numbers of potential patients and associated geographic challenges in the conduct of clinical trials in this population [8]. Furthermore, applying rigid endpoints in rare cancer trials may lead to negative results, underestimating the efficacy of new agents and discouraging the exploration of already approved drugs in treating rare cancers [9]. As a consequence, small studies and case reports are often the only evidence available to guide treatment choices for this group of patients [7].

Patients with rare cancers often experience longer travel times to access specialised cancer care compared to those with common cancers, especially for surgical services and in rural areas [10]. In view of all the above-mentioned challenges, patients with rare cancers are more likely to experience psychological distress, social isolation, and worse quality of life [6, 11]. These issues are exacerbated by the lack of support groups for this population compared to the large and influential groups available for patients with common cancers.

Early-phase clinical trials (EPCTs) may represent a promising treatment option for patients with rare cancers [12]. These trials assess new agents' safety, tolerability and optimal dosing [13]. In recent years, new trial designs such as umbrella and basket studies, the increasing use of molecular profiling to improve patient selection, and the addition of efficacy endpoints (e.g. overall response rate (ORR)) have transformed the EPCT landscape [14]. Access to genomic testing is important to accelerate drug development in rare cancers. Kato et al., performed tissue next-generation sequencing, circulating tumour DNA and protein markers assessment in 40 patients with rare cancers [15]. At least one actionable gene alteration was identified in 92.5% of the patients, while matched treatments were given to approximately half of the patients. In the matched group, the progression-free survival (PFS) was 19.7 months compared to 3.5 months for the previous unmatched treatment (hazard ratio [HR] 0.26, 95% confidence interval [CI] 0.10–0.71, *p* = 0.008).

The current literature reports an unmet need to improve treatment options and prognosis in rare cancers, but also a potentially promising relationship between EPCTs and rare cancers. This single-centre study aimed to assess the role EPCTs could have in the management of rare cancer patients. We also aim to evaluate the use of molecular profiling, the presence of actionable gene alterations and the impact of those in trial selection. Finally, we aim to assess the survival outcomes of patients who participated in EPCTs and compare those who received matched versus unmatched trial treatments.

## Methods

### Study population

A retrospective study was undertaken to evaluate patients with rare tumours referred to the experimental cancer medicine team (ECMT) at the Christie Hospital over five years between 1 January 2016 and 31 December 2020. ECMT runs EPCTs, specifically Phase I and non-randomised Phase II trials for patients with solid tumours and receives referrals for patients that have or nearly have exhausted their standard of care treatment options. The decision to accept a referral is based on patient’s fitness, comorbidities, availability of suitable trials for the specific primary cancer and the opportunity to offer molecular profiling to identify potentially actionable gene alterations. Finally, to consider a patient for this study, they must have had at least one consultation with ECMT.

Rare tumour patients were defined as those tumours with a crude incidence of less than 6 cases per 100,000 per year based on the RARECARENet list of rare cancers and its associated 3-tier system [1, 3]. In this study, we included tumours that were defined as rare based on the tier-1 category. Patients with paediatric cancers were excluded as they are treated in a separate paediatric tertiary centre. Haematological cancers were also excluded as this study focused on solid tumours. Furthermore, epithelial tumours of the cervix were also included despite the tier-1 crude incidence of 6.28. This decision was because all the individual tier-2 categories were rare, and the tier-1 incidence was very close to the rarity margin.

Ethical approval was received from the Quality Improvement and Clinical Audit Committee at the Christie NHS Foundation Trust for the study on 22nd April 2022 (reference 3299).

### Data collection

Data were collected on demographics, medical history, oncological diagnosis and previous treatments (supplementary Table 1). In addition, we documented the sites of metastases, lactate dehydrogenase (LDH) and albumin concentrations to calculate the Royal Marsden Hospital (RMH) prognostic score, which has been validated in EPCTs participants as an independent predictive marker for overall survival (OS) [16, 17]. Similarly, the Gustave Roussy immune score (GRIm score), developed to assist patient selection in immunotherapy-based phase 1 studies, was calculated after collecting LDH, albumin, and the neutrophil-to-lymphocyte ratio (NLR) [18].

In terms of the rare cancer patients' initial consultation with ECMT, we documented three potential outcomes: 1) addition to the active waiting list for trials for patients that were fit and ready to be considered for studies, 2) addition to the watch and wait list for fit patients who were currently receiving other systemic anticancer treatments, and so would not considered for EPCTs until disease progression and 3) discharge to the parent team if the patients were deemed ineligible for EPCTs.

Molecular profiling was offered to selected patients as part of a range of molecular profiling initiatives run within the ECMT with FoundationOne® CDx and FoundationOne® Liquid CDx being the most commonly used genomic testing panels. Profiling was performed predominately as part of the pre-screening processes for specific clinical trials and patient selection was based on each trial’s inclusion and exclusion criteria. In the more recent years, molecular profiling was also offered within the TARGET study, an investigator led study aiming to investigate molecular profiling in potential phase I trial candidates to guide trial selection. Similarly, patients were selected based on the inclusion and exclusion criteria for that trial. When molecular profiling had been performed, we documented the gene alterations identified as well as actionable changes. Actionability was defined as pathogenic variants within specific genes that are targetable by a standard of care treatment or a clinical trial drug. Finally, the cost for molecular profiling was exclusively covered by the relevant trial sponsors.

For the patients offered an EPCT, we collected data on the investigational medicinal products (IMP) mechanism of action and class. The dates for cycle 1 day 1 (C1D1), end of trial visit, progressive disease (PD) or death were recorded, as well as the best response to IMP according to Response Evaluation Criteria in Solid Tumours (RECIST), the reason for discontinuation and duration of participation. For subsequent trial participation, the same data collection was performed for each patient. Finally, we defined PFS as the time from C1D1 to PD or death and OS as the time from the initial ECMT consultation to death from all causes. The follow-up period for data collection ended on 31 May 2022.

### Statistical analysis

Descriptive statistics were performed in all data categories and included central tendency (mean and median), frequency (counts, percentages), and variability (standard deviation (SD)). In univariable analysis of continuous dependent variables, the independent t-test was used, while the Chi-square test was performed for categorical variables. Kaplan–Meier methodology was used for survival analysis and PFS and OS evaluation. Moreover, the Cox proportional-hazards model was used for multivariable survival analysis. The statistics for this study were facilitated by the softwares "IBM SPSS—version 25" and "MedCalc—version 22.023".

## Results

### Demographics

Between 1 January 2016 and 31 December 2020, ECMT received 317 referrals for patients diagnosed with rare cancers. Of those, 77 patients were excluded as they never proceeded to a consultation with ECMT, predominately due to rapid clinical deterioration and poor performance status. Therefore, this study included 240 patients, with 54.2% of the population being females (*n* = 130). The mean age at diagnosis was 52 years, ranging between 16 and 84 years. Females had a lower median age at diagnosis than males (49 vs 57 years), and this relationship was statistically significant (*p* = 0.002) (Table 2).

**Table 2:** Demographic data, medical and oncological history of the studied cohort

Characteristics of rare cancer patients referred to ECMT (2016–2020)	
Characteristic	**Numbers**
Referrals to ECMT	Total: 3118, Rare cancers: 317 (10.2%)
Patients reviewed by ECMT	240 patients
Sex (patients, %)	Males: 110 (45.8%), Females: 130 (54.2%)
Age at diagnosis	Median: 52 years
	Range: 16 – 84 years
Age at diagnosis (males vs females)	Median: 57 years (males), 49 years (females)
Metastasis at diagnosis (patients, %)	• Yes: 98 (40.8%)
	• No: 142 (59.2%)
Metastasis at referral point (patients, %)	• Yes: 207 (86.2%)
	• No: 33 (13.8%)
Number of sites of metastasis (at referral point) (patients, %)	• 0: 33 (13.8%)
	• 1: 96 (40.0%)
	• 2: 78 (32.5%)
	• 3 + : 33 (13.7%)
Time to relapse from initial diagnosis	Median: 13 months, range 1 month – 41 years
ECOG PS at first ECMT consultation (patients, %)	• 0: 43 (17.9%)
	• 1: 168 (70.0%)
	• 2: 24 (10.0%)
	• 3: 5 (2.1%)
Active comorbidities (patients, %)	• 0: 78 (32.5%)
	• 1: 78 (32.5%)
	• 2: 56 (23.3%)
	• ≥ 3: 28 (11.7%)
Family history of any malignancy (non-hereditary) (patients, %)	• Yes: 129 (53.8%)
	• No: 74 (30.8%)
	• Unknown: 37 (15.4%)
Surgery (patients, %)	• Yes: 149 (62.1%)
	• No: 91 (37.9%)
Radiotherapy (patients, %)	• Yes: 132 (55.0%)
	• No: 108 (45.0%)
SACT (patients, %)	• Yes: 190 (79.2%)
	• No: 50 (20.8%)
Lines of SACT (patients, %)	• 0: 50 (20.8%)
	• 1: 129 (53.8%)
	• 2: 45 (18.7%)
	• 3: 15 (6.3%)
	• 4: 1 (0.4%)
	Median: 1 line

*ECMT* Experimental Cancer Medicine Team, *ECOG PS* Eastern Cooperative Oncology Group Performance Status, *SACT* Systemic Anticancer Treatment

### Oncological and medical history

**Fig. 1:**
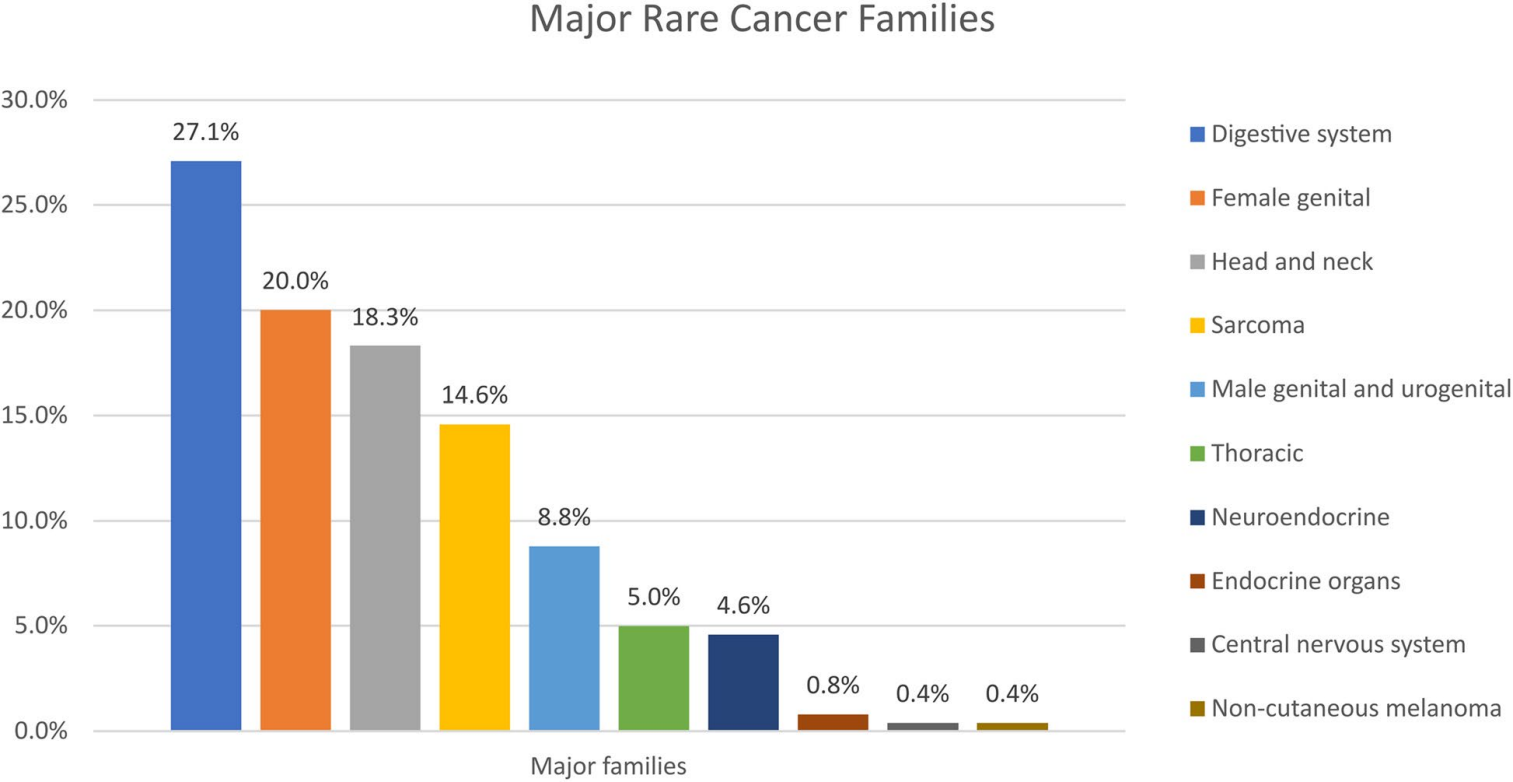
Major rare cancer families of the patient population referred for early phase trials

Digestive system, female genital and head and neck cancers were the commonest tumour types that patients were referred with (major rare cancer families; Fig. 1, tier-1 rare cancer classifications; supplementary Table 2).

De-novo metastatic disease was present in 40.8% (*n* = 98) of the patients, while 86.2% (*n* = 207) had metastatic disease at the time of referral to ECMT. Two-thirds of the patients (72.5%, *n* = 174) had metastases to 1–2 sites, whereas only in 13.7% of the patients three or more metastatic sites were reported. Regarding relapse after initial definitive treatment, the median time to relapse was 13 months, ranging from 1 month to 41 years (a lung neuroendocrine tumour).

As far as previous cancer-related treatments are concerned, 149 patients (62.1%) underwent surgical intervention (either primary resection or metastasectomy), whilst radiotherapy was administered in 45% of the population (*n* = 108). Moreover, SACT had been given to 79.2% of the patients (*n* = 190) with a median number of one prior SACT lines (range 0–4 lines).

### Outcomes of ECMT referrals and molecular profiling analyses

All patients in this study had at least one consultation with ECMT to assess their eligibility for EPCTs. Approximately two-thirds of the patients were actively considered for a clinical trial after this (62.9%, *n* = 151), while 55 patients (22.9%) were currently receiving standard of care SACT during the initial consultation, so unsuitable for early phase trial recruitment at that point. Finally, 14.2% of the population (*n* = 34) were assessed as ineligible for clinical trials based on poor ECOG PS, significant comorbidities or deranged blood tests.

Molecular profiling was performed by evaluating archival tumour samples (77%) or circulating tumour DNA (23%) in 45.5% of our population (109 patients). This has been increasingly offered over the studied period, with a significant rise from 2016 (18.8%) to 2019 (54.5%). At least one gene alteration was reported in 75.2% (*n* = 83) of the patients who underwent profiling, while the median number of alterations was three (range 1–20). The most frequently mutated genes were TP53 (43.4%, *n* = 36), PIK3CA (24.1%, *n* = 20), KRAS (15.7%, *n* = 13) and CDKN2A (12.0%, *n* = 10).

**Fig. 2:**
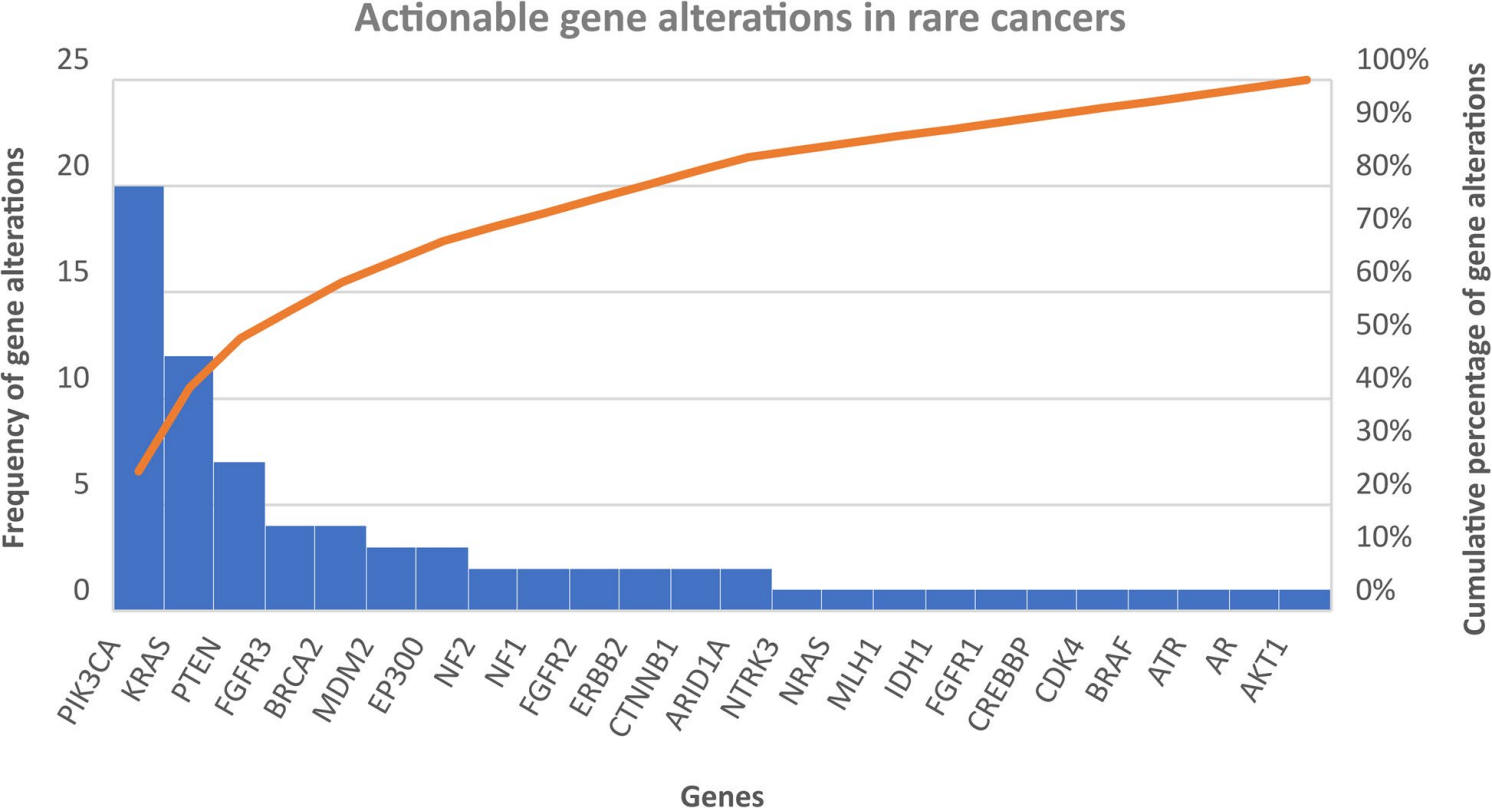
Bar chart demonstrating the genes reported with actionable alterations and line graph showing the cumulative percentage of gene alterations in patients with rare cancers

Actionable gene alterations were reported in 50 patients, representing 45.9% of those with molecular profiling and 60.2% with identified gene alterations. PIK3CA, KRAS and PTEN were the genes in which actionable aberrations were most commonly identified (Fig. 2). The digestive system (*n* = 21, 32.3% of this tumour group), followed by sarcomas (*n* = 9, 25.7% of this tumour group) and head and neck tumours (*n* = 7, 15.9% of this tumour group), were the major rare cancer families with the highest number of patients with actionable alterations.

### Participation in EPCTs

EPCTs, both matched and unmatched, were offered to 54 patients in the studied population. Three patients failed to meet the required eligibility criteria after screening; therefore, 51 patients proceeded with a trial. There has been a steady increase in rare cancer patients participating in ECMT studies from 2016 to 2019, while 2020 was an exception due to reduced trial recruitment secondary to the COVID-19 pandemic. Female genital (*n* = 15, 33.3%), NET (*n* = 3, 27.3%), digestive system (*n* = 15, 23.1%) and head and neck (*n* = 10, 22.7%) cancers were the major families which reported the highest percentages of trial participants within each family. Regarding IMP classes, 49% of the patients (*n* = 25) received targeted treatments, and 35.3% (*n* = 18) had immunotherapeutic agents. Chemotherapy-based agents, antibody–drug conjugates and combination treatments were other IMP classes investigated in ECMT trials. Approximately one-third of trial participants (29.4%, *n* = 15) enrolled in matched trials based on the presence of gene aberration.

Overall, the median duration of participation in EPCTs was two months, ranging from 2 weeks to 34 months. In terms of best responses, 47.1% of the participants showed PD, 39.2% demonstrated stable disease (SD) with a median duration of 6 months (SE 0.74, 95% CI 4.55 – 7.45), and partial response (PR) was achieved by 11.8% of the patients with a median duration of 13 months (SE 4.90, 95% CI 3.40 – 22.60). Moreover, the median PFS for trial participants was three months (95% CI 1.12 – 4.88). PD was the main reason for trial discontinuation (62.7%), followed by adverse events (27.5%). Finally, four patients participated in a second EPCT, with the median duration of participation being 14 months, and SD was achieved in half of them (supplementary Table 3).

### Clinical outcomes and survival analysis

The median follow-up time for this cohort was 8 months. From the studied population, 211 deaths occurred by the end of the follow-up date on 31 May 2022. Median OS was 8 months (95% CI 6.93 – 9.07), while 12-month and 3-year OS were 32% and 11.9%, respectively.

**Fig. 3:**
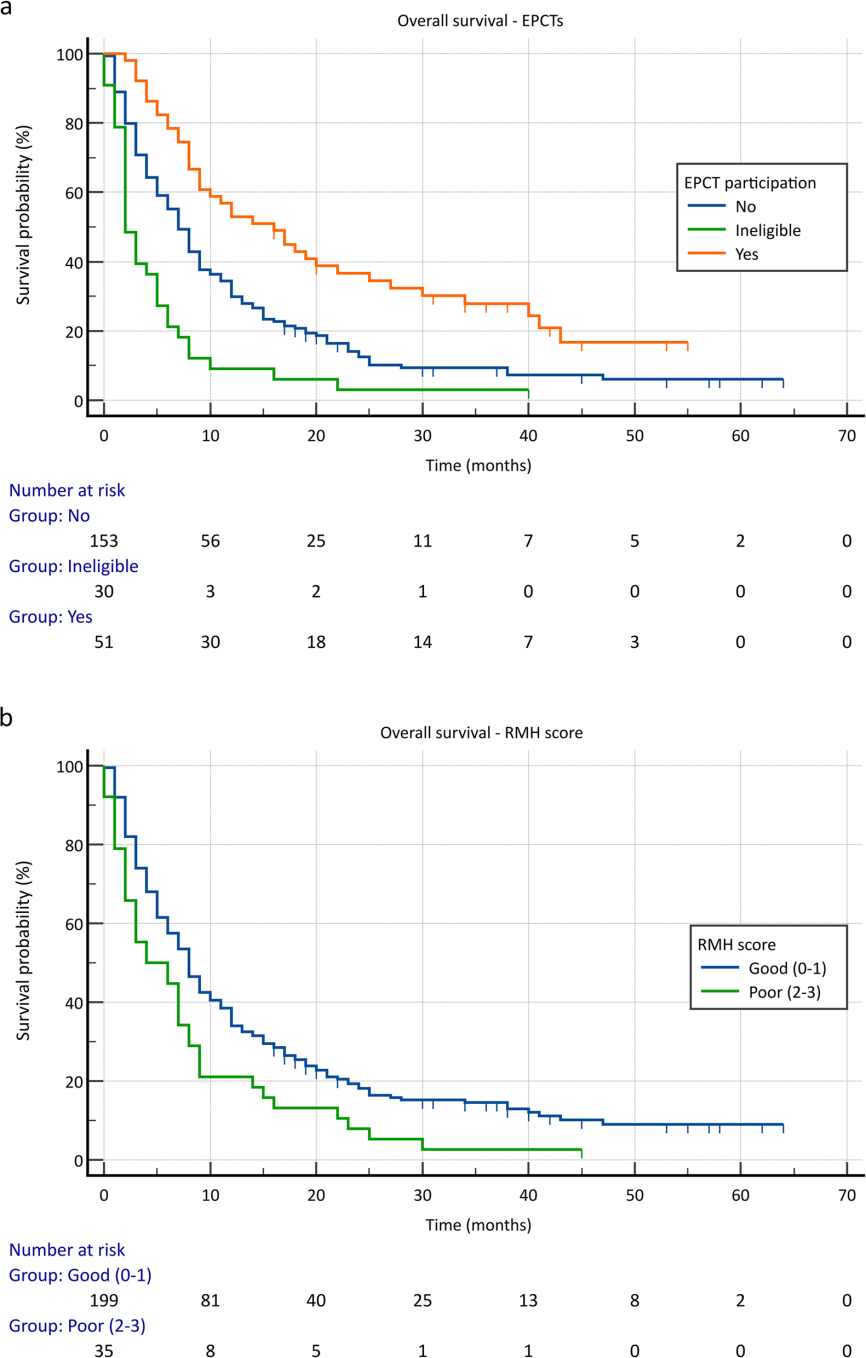
**a** OS comparison of EPCTs participants vs fit patients who did not proceed with a trial vs ineligible patients, **b** OS comparison between patients with good (0–1) and poor (2–3) RMH predictive scores

For EPCT survival analysis, the population was split into three categories: 1) eligible patients who were offered an ECPT, 2) patients who were deemed suitable for EPCTs but did not participate in a study for reasons such as lack of trials aiming at the reported actionable abnormalities or lack of appropriate trial slot availability and 3) patients ineligible for trials. The analysis demonstrated a clear survival benefit for the trial population with a median OS of 16 months (95% CI 9.10 – 22.90) compared to 7 months for non-trial participants (95% CI 5.50 – 8.51), and 2 months for ineligible patients (95% CI 1.13 – 2.87) (*p* < 0.001) (Fig. 3a). Finally, in terms of the outcomes of the patients that received matched trial treatments versus those that participated in unmatched studies, there was no statistically significant OS difference seen (*p* = 0.96).

We assessed the relationship between RMH and GRIm scores with OS in rare cancer patients. Patients with good RMH scores (0–1) demonstrated a median OS of 8 months (95% CI 6.61 – 9.38) compared to 4 months for those with poor RMH scores (2–3) (95% CI 0.00 – 8.53) (*p* = 0.007) (Fig. 3b). Similarly, a low GRIm score (0–1) was associated with a median OS of 8 months (95% CI 6.43 – 9.57) vs 4 months for a high GRIm score (2–3) (95% CI 2.13 – 5.88) (*p* < 0.001). We also performed a multivariable Cox regression, including sex, age category at diagnosis (≤ 50 years vs > 50 years) and the RMH score. The analysis demonstrated that patients with poor RMH score had a 71.4% increased risk of death compared to those with a good RMH score when controlling for the age category and sex (HR 1.714, 95% CI 1.19 – 2.46, *p* = 0.004).

## Discussion

Rare cancers comprise a major public health issue and significantly impact the general population, accounting for at least 20% of all cancer diagnoses [1]. This burden is often underestimated as patients with rare cancers have limited treatment options, scarce research opportunities and significantly decreased number of support groups, as described earlier. This study identified 317 rare cancer patients, representing approximately 10% of all cancer patients referred to ECMT (*n* = 3118). Compared to the literature, this smaller percentage can be partly explained by the exclusion of patients with haematological and paediatric tumours. Another possible reason may be that fewer patients with rare cancers are being referred to ECMT due to rapid clinical deterioration and/or lack of awareness of EPCTs by the parent treating teams.

In this study, patients with rare cancers from the digestive system, female genital and head and neck cancers were the most frequently referred, a finding that was also observed in another two major studies [1, 2]. Additionally, in our population, the endocrine organ tumours, CNS cancers and, skin cancers and non-cutaneous melanomas shared the lowest number of patients, while in the study of Gatta et al., embryonal tumours, skin cancers and non-­cutaneous melanomas were the least frequent families [1]. Our figures were likely to be influenced by the specific referral patterns and disease expertise in our cancer centre in addition to reflecting tumour biology, trial availability and patients' fitness.

EPCTs have undergone a significant transformation over the past two decades, with increased response rates reported in many early phase trials now, compared to historical trials [12]. On average, ORRs of 20% were noted in a review of contemporary EPCTs [19]. In EPCTs where the patient selection is based on genomic biomarkers, ORR can increase to 42% [20]. Furthermore, drug-related mortality in phase I studies, testing single-agent IMPs, remains low at < 1% [21]. These data support the hypothesis that EPCTs could be a valid treatment option, especially for rare cancer patients given the lack of standard of care systemic therapies licenced for many rare tumours. Our study described an increasing number of patients being recruited to EPCTs each year (2016–2019) apart from 2020 potentially due to altered referral/recruitment pathways in the COVID-19 pandemic.

Precision medicine has been a significant contributor to EPCTs transformation [12, 14]. In this study, molecular profiling was offered to almost half of the population and this smaller percentage can be explained by the exclusion of ineligible patients (*n* = 34) and the limited access to profiling through trial routes in the early years of the studied period. In the 45.9% of the patients that underwent profiling, at least one actionable gene alteration was identified and could guide trial selection. Overall, all-comers trials are being replaced by newer designs such as the basket studies, in which the patients are selected based on the presence of specific gene alterations rather than the primary tumour [22]. This tumour agnostic drug development approach can be an important route to clinical trials for rare cancer patients. A characteristic example is larotrectinib which has been approved for patients with TRK fusion–positive cancers irrespective of the tumour type [23]. Interestingly, in this study, 74.5% of the participants were patients with rare cancers.

The overarching aim of this study was to assess the effect of participation in EPCTs on the clinical outcomes of patients with rare cancers. It is promising that we showed a median OS benefit of 9 months for the trial participants compared to the patients for whom no trial was identified study despite being assessed as suitable for trials. To our knowledge of the current literature, this is the first study, although non-randomised, which demonstrates a survival benefit in rare cancer patients enrolled in EPCTs. Unfortunately, 64.3% (*n* = 97) of the patients that were actively considered for a clinical trial during the initial consultation, were not offered a trial despite being fit mainly because no suitable trial was identified, or trial slot availability was limited. For these patients, the lack of trial options and the fact that they had exhausted standard of care treatments would inevitably lead to disease progression and shorten their survival. Moreover, only one in three patients (*n* = 15) with detected actionable aberrations participated in an EPCT. Given the potential benefits of recruitment to EPCTs, increasing the available trial options for patients with rare tumours is therefore a critical area for focus in the future.

We evaluated the RMH prognostic score as an independent OS marker for patients with rare cancers, demonstrating a median OS of 8 months for patients with good RMH score compared to 4 months in patients with poor RMH score. Moreover, the previously mentioned multivariate analysis, including RMH score, age category and sex, showed that a good RMH score remains associated with a significantly reduced risk of death. The RMH score could be implemented into day-to-day practice to assist with the appropriate selection of rare cancer patients for EPCTs.

There are limitations to this study. The conflicting definitions for rare cancers used in the past made comparing our results to the literature sometimes difficult. Our population was also a heterogeneous group of patients, including tumours from ten different major families; hence, a more in-depth analysis of each tumour group was precluded. Furthermore, this was a retrospective 5-year study; understandably, some patient data were missing while the COVID-19 pandemic negatively affected referral numbers and limited trial options for patients in 2020.

In order to curb the rising incidence and improve survival outcomes for patients with rare cancers, several interventions could be explored. Educating healthcare professionals and raising public awareness can lead to earlier diagnosis [6, 24], and a unified worldwide definition of rare cancers would be necessary for everyday clinical practice and research as it would allow direct comparisons between datasets. Indeed, up-to-date cancer databases would be valuable in monitoring the incidence of rare cancers and driving the development of robust guidelines and further research in this field. Centralising care for rare cancers is particularly important to decrease the disparities with common cancers, while it has been correlated with improved outcomes [6, 25]. Centres of reference offer multidisciplinary teams of experts and can access novel treatments leading to superior overall management [1].

Participation of patients with rare cancers in research should be encouraged as this would be the pathway for developing new lines of treatment. As underscored in our study, EPCTs are a promising opportunity, and participation of patients diagnosed with rare cancers should be encouraged. Molecular profiling can offer vital information for treatment selection in rare cancers and can increase trial options by providing access to agents being developed in a tumour agnostic fashion [26]. Therefore, it should become more widely available for patients. In that direction, DETERMINE is the first UK trial using a precision medicine platform to match rare cancer patients with known molecular alterations to already licenced treatments for other, mainly common cancers [27].

## Conclusions

Overall, rare cancers carry a significant burden, accounting for 20% to 24% of all cancers in the US and Europe, respectively. The incidence of rare cancers has been increasing while survival outcomes remain significantly worse compared to common cancers. Rare cancer patients experience delayed diagnosis, scarce treatment options, reduced opportunities for participation in research, and a lack of support groups.

EPCTs represent a viable potential treatment option for rare cancer patients as they have undergone significant transformation with the inclusion of efficacy endpoints. In this non-randomised study, we showed, a survival benefit for EPCT participants with rare cancers. However, we also demonstrated that the lack of trial options remains a significant barrier for patients with rare cancers, requiring concerted efforts from the research community going forwards. Nevertheless, the wider use of molecular profiling is anticipated to increase trial options and for these patients. Ultimately, the aim of these interventions is to enhance future research initiatives to ultimately deliver new treatments for rare cancer patients.

## Supplementary Information

Supplementary Material 1: Table 1. Detailed description of data categories collected on the studied cohort. ECMT; Experimental Cancer Medicine Team, ECOG PS; Eastern Cooperative Oncology Group Performance Status, EPCT; Early Phase Clinical Trial, IMP; Investigational Medicinal Product, SACT; Systemic Anticancer Treatment, PFS; Progression Free Survival, OS; Overall Survival. Table 2. Description of the tier-1 categories of patients referred for early phase clinical trials (EPCTs). Table 3. Clinical data of the participants enrolled onto early phase clinical trials (EPCTs).

## Data Availability

The datasets used and/or analysed during the current study are available from the corresponding author on reasonable request.
