# Supplementary material for: A single centre experience of patients with rare cancers referred for early phase clinical trials
Source: BMC Cancer. 2025 Mar 28;25:558. doi: 10.1186/s12885-025-13934-2 (PMC11951660; doi:10.1186/s12885-025-13934-2)
Supplement: Supplementary file 1 — Supplementary Material 1: Table 1. Detailed description of data categories collected on the studied cohort. ECMT; Experimental Cancer Medicine Team, ECOG PS; Eastern Cooperative Oncology Group Performance Status, EPCT; Early Phase Clinical Trial, IMP; Investigational Medicinal Product, SACT; Systemic Anticancer Treatment, PFS; Progression Free Survival, OS; Overall Survival. Table 2. Description of the tier-1 categories of patients referred for early phase clinical trials (EPCTs). Table 3. Clinical data of the participants enrolled onto early phase clinical trials (EPCTs). [file 12885_2025_13934_MOESM1_ESM.docx]

**Supplementary tables**

| Collected data on the studied population | |
| --- | --- |
| Categories | **Details** |
| Sex |  |
| Age | At time of ECMT consultation |
| Date of diagnosis | Initial diagnosis with cancer |
| Date of death |  |
| Date of relapse | First relapse post initial diagnosis (excludes patients with de novo metastatic disease) |
| Time to relapse | The length of time from initial cancer diagnosis to first radiological evidence of disease relapse |
| Major rare cancer families | As per the Joint Action on Rare Cancers |
| Tier-1 categories | As per the RARECARE classification |
| Histological diagnosis of tumour |  |
| RMH prognostic score | Sites of metastases, albumin, LDH |
| GRIm score | Neutrophil-to-lymphocyte ratio, albumin, LDH |
| ECOG PS | ECOG Performance status as assessed at time of ECMT consultation |
| Comorbidities | Actively affecting the patients at the time of ECMT Consultation |
| Family history of malignancies | Record of the presence of any type of malignancy in relatives |
| Previous treatments | Surgery, radiotherapy, SACT |
| Lines of SACT | At the time of ECMT referral |
| Outcome of ECMT consultation | Active waiting list, watch and wait, ineligible for EPCTs |
| Molecular profiling | Number of patients in whom it was performed, gene alterations, actionable aberrations |
| Participation in early phase trial | IMP (mechanism of action, class of agent), C1D1, end of trial visit, duration of participation, best response, reason for discontinuation, subsequent trials |
| PFS | The length of time from start of treatment to disease progression or death |
| OS | The length of time from first ECMT consultation to death |

*Supplementary table 1: Detailed description of data categories collected on the studied cohort. ECMT; Experimental Cancer Medicine Team, ECOG PS; Eastern Cooperative Oncology Group Performance Status, EPCT; Early Phase Clinical Trial, IMP; Investigational Medicinal Product, SACT; Systemic Anticancer Treatment, PFS; Progression Free Survival, OS; Overall Survival.*

| Major rare cancer families and tier-1 categories in the studied cohort | |
| --- | --- |
| Overall number of cases | - 240 patients |
| Head and neck tumours (patients, %)  N = 44 | - Salivary glands: 19 (43.2%) - Oropharynx: 13 (29.5%) - Sinuses: 5 (11.4%) - Hypopharynx: 3 (6.8%) - Nasopharynx: 3 (6.8%) - Oral cavity: 1 (2.3%) |
| Digestive system tumours (patients, %)  N = 65 | - Extrahepatic biliary ducts: 25 (38.5%) - Anal canal: 15 (23.1%) - Gallbladder: 14 (21.5%) - Small intestine: 11 (16.9%) |
| Thoracic tumours (patients, %)  N = 12 | - Malignant melanoma: 10 (83.3%) - Thymoma: 2 (16.7%) |
| Female genital tumours (patients, %)  N = 48 | - Cervix: 40 (83.3%) - Vulva: 6 (12.5%) - Vagina: 2 (4.2%) |
| Male genital and urogenital tumours (patients, %)  N = 21 | - Penis: 11 (52.4%) - Ureter: 5 (23.8%) - Renal pelvis: 4 (19.0%) - Testis: 1 (4.8%) |
| Skin cancers and non-cutaneous melanoma (patients, %)  N = 1 | - Mucosal melanoma: 1 (100%) |
| Sarcomas (patients, %)  N = 35 | - Soft tissue sarcoma: 32 (91.4%) - Bone sarcoma: 3 (8.6%) |
| Neuroendocrine tumours (NET) (patients, %)  N = 11 | - NET other sites: 5 (45.6%) - NET gastrointestinal pancreatic: 3 (27.2%) - NET lung: 3 (27.2%) |
| Endocrine organ tumours (patients, %)  N = 2 | - Thyroid: 2 (100%) |
| Central nervous system (CNS) tumours (patients, %)  N = 1 | - Malignant meningioma: 1 (100%) |

*Supplementary table 2: Description of the tier-1 categories of patients referred for early phase clinical trials (EPCTs).*

| EPCTs participants clinical data | |
| --- | --- |
| Number of participants | 51 patients |
| Major rare cancer families (patients, %) | - Digestive system: 15 (29.4%) - Female genital cancers: 15 (29.4%) - Head and Neck cancers: 10 (19.6%) - Neuroendcrine tumours: 3 (5.9%) - Sarcomas: 3 (5.9%) - Male genital and urogenital tumours: 3 (5.9%) - Thoracic tumours: 1 (1.95%) - Endocrine organ tumours : 1 (1.95%) - Skin cancers and non-cutaneous melanomas: 0 (0%) - Central nervous system tumours: 0 (0%) |
| Investigational medicinal product classes (patients, %) | - Targeted agents: 25 (49.0%) - Immunotherapy: 18 (35.3%) - Antibody drug conjugate: 4 (7.8%) - Chemotherapy: 3 (5.9%) - Combination (targeted + immunotherapy): 1 (2.0%) |
| Matched vs unmatched studies (patients, %) | - Matched: 15 (29.4%) - Unmatched: 36 (70.6%) |
| Duration of participation (months) | - Median: 2 months - Range: 0.5 - 34 months |
| Best response (patients, %) | - Progressive disease: 24 (47.0%) - Stable disease: 20 (39.2%) - Partial response: 6 (11.8%) - Complete response: 1 (2.0%) |
| Reason for discontinuation (patients, %) | - Progressive disease: 32 (62.7%) - Adverse events: 14 (27.5%) - Clinical deterioration: 3 (5.9%) - Ongoing: 2 (3.9%) |
| Progression free survival | - Median: 3 months (95% CI 1.12 – 4.88) |
| Subsequent participation in EPCTs (patients) | - Second trial: 4 - Third trial: 1 |

*Supplementary table 3: Clinical data of the participants enrolled onto early phase clinical trials (EPCTs).*
